# A survey of local health promotion initiatives for older people in Wales

**DOI:** 10.1186/1471-2458-8-217

**Published:** 2008-06-20

**Authors:** Maggie Hendry, Nefyn H Williams, Clare Wilkinson

**Affiliations:** 1 Department of Primary Care and Public Health, Cardiff University, North Wales Clinical School, Wrexham, UK; 2 Department of Primary Care and Public Health, Cardiff University, North Wales Clinical School, Gwenfro Unit 6/7, Wrexham Technology Park, Wrexham, LL13 7YP, UK

## Abstract

**Background:**

As the demographic profile of the UK changes, policy makers and practitioners have to respond to health challenges presented by a progressively ageing population. The health promotion plan for older people, aged over 50 years, in Wales included eight key areas: physical activity, healthy eating, home safety and warmth, emotional health, health protection, smoking, alcohol and sexual health. The aim of this study was to describe the extent, content and regional variation of existing health promotion initiatives for older people in Wales, provided by statutory, voluntary and private sector agencies.

**Method:**

A questionnaire was sent to senior health promotion specialists employed in the 22 local authority areas in Wales to ascertain details of all projects promoting health and wellbeing in the eight key areas where the priority population was aged over 50, or the majority of users were older people. Additional information was sought from project leads and websites.

**Results:**

Eighteen questionnaires were returned; not all were fully completed. Four areas did not return a questionnaire. Additional information was obtained from internet searches but this mainly concerned national initiatives rather than local projects. In all, 120 projects were included, 11 were throughout Wales. Best provision was for physical activity, with 3 national and 42 local initiatives, but local provision was patchy. Healthy eating, and home safety and warmth had far fewer initiatives, as did health protection, which comprised two national immunisation campaigns. Smoking and alcohol misuse were poorly provided for, and there was no provision for older people's sexual health. Evaluation arrangements were poorly described. Half of those who responded identified unmet training needs.

**Conclusion:**

The reasons for patchy provision of services were not clear. Increased efforts to improve the coverage of interventions known to be effective should be made. Rigorous evaluation of projects is needed to ascertain the most effective and appropriate interventions, especially for alcohol misuse and sexual health. These conclusions are relevant to the other countries of the United Kingdom (UK), and more widely across Europe.

## Background

Due to a combination of decreasing birth rates and increasing longevity, the population of Europe is ageing and the proportion of older people (aged 50 or over) is predicted to rise to 44% by 2025 [1]. In Wales 37.2% of the population are currently over 50, with a projected increase to 43% by 2025 [2]. The report, 'Healthy Ageing: a Challenge for Europe' [3], aimed at policy makers and practitioners in European Union (EU) member states, identified the following priority topics for health promotion: retirement and pre-retirement, social capital, mental health, environment, nutrition, physical activity, injury prevention, substance use/misuse (including smoking and alcohol), medication and associated problems, and preventive health services. Welsh Assembly Government priorities for older people [4,5] include the middle-aged population over 50 years old and are broadly similar. The first three priorities are included in the area of emotional health; environment and injury prevention partly overlap with home safety and warmth; medication is not included, and there is an additional priority topic, sexual health. The Welsh priorities are as follows :

• Physical activity

• Healthy eating

• Home safety and warmth

• Emotional health

• Immunisation

• Smoking

• Alcohol

• Sexual health

In 2006 the Welsh Assembly Government commissioned a survey to assess the coverage of health promotion activities in these eight priority areas across Wales.

## Aim

The aim was to describe the extent, content and regional variation of existing health promotion initiatives for older people in Wales, provided by statutory, voluntary and private sector agencies in order to:

• Provide a baseline against which to measure any increase in activity

• Identify areas of promising practice to disseminate to other areas and other agencies in Wales

• Identify any major training needs in relation to promoting the health of older people

## Method

A specification for the survey was produced by the policy lead for the Health Promotion Division of the Welsh Assembly Government (the Client). The method for conducting the survey was developed through a process of consultation with the Client during which a draft questionnaire was presented for comments and amended until a final version was agreed. The questionnaire asked about projects in each of the eight priority areas and other projects outside of these priorities. The following information was asked for each project: project title, aims, whether part of an over-arching health promotion programme for older people, organisations involved, setting, geographic scope, target group, number of clients involved, evidence base and evaluations. In addition the questionnaire asked about training needs for the providers of the projects and gave instructions for the completion and return of the questionnaire. See Table 1 for an abbreviated version of the questionnaire or, for the full version, see Additional file 1.

**Table 1:** Questionnaire about local health promotion programmes and projects for older people.

**Section 1 Projects that promote physical activity**	
e.g. encouraging increased activity; participation in sports and exercise.	
	
**What is the project title?**	
	
**What is the overall aim of the project?**	
	
**Is the project part of an overarching health promotion programme for older people?**	Yes/No
If yes, what is the strategic context of the programme? e.g. Health, Social Care and Well-being Strategy or Health Alliance Strategy.	
	
**Organisation(s) involved**	
Give name(s) and indicate whether statutory, voluntary or private sector agency. If one or two agencies lead the project, please specify which one(s) and give details of the main contact person in each.	
	
**Setting**	
e.g. the home; leisure centre or other community setting, primary care, secondary care, intermediate care; residential or nursing home.	
	
**Geographic scope of project**	
e.g. is the project throughout the local authority, or is it confined to specific communities or institutions?	
	
**Target group**	
Please give a brief description of any defining characteristics of the target group, for e.g. age, gender, social circumstances, health status if relevant (e.g. overweight, at high risk of CHD, has sustained a previous fall)	
	
**Numbers involved**	
Please give an estimate of the number of people in the target group who are currently involved in the project... ...and the maximum capacity of the project.	
	
**Evidence base**	
Is the project informed by evidence, eg guidelines, national service framework standards?	Yes/No
If yes, please say what evidence was used.	
	
**Evaluation**	
Are processes in place for monitoring or evaluation of the project?	Yes/No
If yes, please describe them.	
	
**Section 10 Training needs**	
This section applies to all the projects described in this questionnaire.	
	
In the course of developing or running these projects, have you become aware of any training needs in relation to health promotion for older people?	Yes/No
	
If yes, what were they and how have they been met?	
	
If the training needs could not be met, please give reasons. e.g. were no appropriate courses available, or was there a lack of funding to pay for training?	

In consideration for space, only the questions are given here; other information such as instructions for completion and return are omitted. The questions were repeated for each of the eight topic areas and for "other projects" (if any) that did not fit the specific topic areas

### Inclusion criteria for projects

• Projects where the priority population were aged over 50 or where the majority of users were older people.

• Projects promoting health and wellbeing in the categories: exercise, healthy eating, home safety and warmth, emotional health, immunisation, smoking, alcohol and sexual health.

### Exclusion criteria for projects

• Schemes addressing existing ill-health (e.g. cardiac rehabilitation)

• Projects where the priority population was not older people.

• Projects whose main aim was not health promotion (e.g. adult education schemes)

• Projects where insufficient information was available to make a useful report or to determine whether the aim was health promotion or the priority population older people (e.g. only project title given)

The target group for the questionnaire, referred to as the Primary Informants (PIs) were senior health promotion specialists in the local public health departments in each of the twenty two local authorities in Wales, who were identified by the Client. An electronic and postal questionnaire was sent to each of them with a covering letter signed by the Client. The PIs could complete it by hand or electronically, and return it by post or e-mail, according to their preference. Two e-mail reminders were sent to non-responders after two and four weeks, followed by a telephone contact. PIs who still failed to respond were contacted again by the Client. Data were entered onto a Microsoft Access database. The 22 local authorities were referred to by the letters A to V.

The questionnaire asked PIs to give the name and contact details for the person responsible for each project named in the questionnaire so that these Secondary Informants (SIs) could be asked to provide supplementary information. Where data were missing, SIs were contacted by telephone or e-mail. Additional projects that had not been identified in the completed questionnaire but were revealed by the SI were included and, if necessary, third parties for those projects were contacted. Further information was retrieved from the websites of organisations responsible for projects. Further information was not sought from projects that comprised local implementation of a Wales-wide programme.

Questionnaire data were extracted for each priority area. Separate tables were constructed for Wales-wide initiatives and local projects. We attempted to quantify local initiatives according to their provision within the local authority area. Small projects were only in one area or served fewer than 50 people; medium – sized were in more than one location and served more than 50 people; large projects served people throughout the local authority area.

## Results

### Questionnaire completion and return

Eighteen questionnaires were returned. The degree of completion varied; none were fully completed. Where PIs were unable to complete sections but had given details of SIs, efforts were made to contact them by telephone or email so that gaps could be filled. Four local authority areas, A, D, G and M, did not return a questionnaire. These areas were spread across Wales and included a mix of urban, rural, deprived and affluent populations. Some information was obtained from an internet search, but this mainly concerned national initiatives, rather than local projects in these areas.

### Health promotion initiatives reported

One hundred and twenty projects were included, 11 were throughout Wales; 49 were excluded because they did not meet the inclusion criteria (Table 2).

**Table 2:** Number of health promotion initiatives reported by topic

**Topic Area**	**Wales-wide programmes**	**Regional or local projects**
Physical activity	3	42
Healthy eating	1	14
Home safety & warmth	1	19
Emotional health	3	11
Health protection	2	4
Smoking	0	1
Alcohol	0	1
Sexual health	0	0
Other initiatives	1	17

**Total**	**11**	**109**

**Grand total**	**120**	

#### Physical activity

The largest number of health promotion activities for older people were in this category, including three Wales-wide programmes and 42 regional or local projects. Free swimming was available for people aged 60 and over in all local authority run swimming pools. The EXTEND programme used gentle exercise to music in residential care settings, but although there were EXTEND teachers throughout Wales their numbers were reported to be insufficient to meet demand. There were guided walk schemes in all areas and, whilst these were open to all ages, it was reported that participants tended to be over 50. Physical activity formed one strand of the national Keep Well This Winter (KWTW) campaign but this mainly involved advice and information. The Moving More Often programme, aimed at frail older people, was being piloted in six areas with a view to being rolled out across Wales if it proved to be effective. Provision was unevenly spread across Wales, with five local initiatives reported in areas K, O and U, but none in areas E, F and Q. No physical activity initiatives were reported that targeted older members of an ethnic minority group.

#### Healthy eating

Lunch clubs were widespread throughout Wales, but little information was available about the extent to which the food provided was 'healthy', or whether the opportunity was taken to promote healthy eating messages. Healthy eating formed another strand of the KWTW campaign's 'Keep Well' theme and healthy eating stands with information and cookery demonstrations were included in KWTW road shows. Healthy eating initiatives were reported in only nine local authority areas, and nine of the 14 projects reported were small. Show and tell methods (events, talks, demonstrations, leaflets etc) were most commonly used; only four projects worked closely with groups of people over a number of sessions offering 'hands on' experience to improve cooking, food shopping or budgeting skills. In a few areas initiatives such as Green Gyms and food co-operatives attempted to improve access to locally produced seasonal fruit and vegetables, though not exclusively for older people.

#### Home safety and warmth

Home safety and warmth formed key elements of KWTW's 'keep warm' and 'keep safe' themes, mainly addressed by 'safe and sound' road shows, featuring a number of stands and demonstrations including home safety, energy efficiency, electric blanket testing, free low energy light bulbs and smoke alarms, advice about home heating, improvement grants, help with bills and avoiding falls. The charitable organisation Care and Repair Cymru operated throughout Wales providing home improvements, repairs and adaptations to help prevent accidents and falls and could recommend reputable and reliable tradespersons. Local initiatives addressed fuel poverty and related illness, falls prevention, or home safety. Provision was patchy with 19 projects in 10 areas. Eight local projects aimed to reduce falls, but half of the se only involved the distribution of 'non-slip' slippers.

#### Emotional health

Emotional health initiatives promoted social interaction, befriending and counselling, and intellectual stimulation. The University of the Third Age provided a wide range of activities encouraging social interaction, intellectual stimulation and physical activity, Age Concern's Better advice: Better Health scheme focussed on welfare and benefits advice, and the Community Service Volunteers' Retired and Senior Volunteer Scheme gave older people the opportunity and training to undertake voluntary work in a variety of settings. Age Concern operated befriending schemes (areas K, L and R); a counselling service that addressed crisis issues such as adjusting to retirement, bereavement, anxiety, family and relationship problems (areas K and L); and welfare and benefits advice (area B).

Initiatives to engage older people in creative activities such as arts and crafts were not well reported, possibly because such initiatives might not be seen within the remit of health promotion specialists. Opportunities existed nationally within the University of the Third Age; the only local initiative reported was an older persons' Eisteddfod (competitions in music, arts and crafts) in a district of area L. A social element was incorporated into many local initiatives such as lunch clubs.

#### Health protection

The main health protection measures we re the nation-wide influenza and pneumococcal immunisation programmes. An annual KWTW campaign aimed to maximise uptake. In addition four local projects in three areas promoted immunisation and general health awareness, and two of them provided health screening (BP, cholesterol, BMI etc).

#### Smoking cessation

Area L planned a review of smoking cessation services for older people. Area H Smoking Cessation Services manned a stand at an event for older people (KWTW 2004/5 campaign). There were no other reports of smoking cessation services that specifically targeted older people or were adapted to meet their needs.

#### Sensible drinking

There were no reported health promotion initiatives that explicitly took alcohol consumption into account, either in terms of the causes of drinking, or its effects (falls and accidents, malnutrition, confusional states). No initiatives were described that encouraged people to recognise and seek help for their problem drinking, or to enable family members or care workers to identify the problem and to respond appropriately. There was only one local authority area (K) whose alcohol and drug abuse services had specialist treatment workers to help older people manage their alcohol consumption.

#### Initiatives that promote sexual health

No initiatives promoting the sexual health of older people were reported.

### All health promotion initiatives: intensity of provision

The quantification of health promotion provision was complicated by variation in: the quality of questionnaire completion; geographical spread of projects from local schemes to Wales-wide initiatives; numbers of people involved; project duration; different intensity of contact with the target group; age range; additional eligibility criteria such as ethnicity, location or accommodation type.

#### Wales-wide initiatives

A substantial amount of health promotion activity in Wales is associated with nation wide initiatives (Table 3, including initiatives that are not specifically for older people but which may be appropriate for older people to use). There are initiatives to address all areas of health promotion except for sexual health.

**Table 3:** Wales-wide programmes that are available to older people and other adults

	Physical exercise	Healthy eating	Safety & warmth	Emotional health	Health protection	Smoking	Alcohol	Sexual health
Alcohol Concern							✓	
Alcoholics Anonymous							✓	
Better Advice: Better Help (welfare & benefits)				✓				
Breast Test Wales					✓			
Care and Repair			✓					
Drinkline (free 24 hour helpline)							✓	
Exercise referral schemes	✓							
EXTEND	✓							
Flu vaccination programme					✓			
Free prescriptions					✓			
Free sight tests for the over 60s					✓			
Free swimming for the over 60s	✓							
Keep Well this Winter	✓	✓	✓		✓			
Lunch Clubs		✓		✓				
National smoking cessation service						✓		
Pneumococcal vaccination programme					✓			
Retired and Senior Volunteer Programme				✓				
University of the Third Age				✓				
Walking the Way to Health	✓							

#### Local initiatives

Initiatives were classified as small, medium and large to indicate the provision of local health promotion initiatives by local authority area (Table 4). There was no consistent pattern can be observed but area L, which had the greatest number of people aged over 50, had the greatest intensity of provision. This might also be because the area L PI returned the most fully completed questionnaire. By contrast area S, which had the largest surface area, the sixth largest population and the second highest proportion of older people, had relatively few local health promotion initiatives for older people. Areas E and F reported no local initiatives at all.

**Table 4:** Intensity of local health promotion provision by local authority

**Local authority**	**Number aged 50+ (% of total population)**	**Physical activity**	**Healthy eating**	**Home safety & warmth**	**Emotional health**	**Health protection**	**Smoking**	**Alcohol**	**Sexual health**	**Other projects**
L	89,396 (29.3%)	▢□□	▢■		□□■■					▢□□
H	81,359 (36.4%)	▢▢	▢				▢			
G	79,566 (34.3%)	No data available from primary informant								
R	68,363 (39.4%)	▢□	□		□	□				
N	56,623 (33.4%)	▢	■			■				
S	51,299 (40.6%)	▢□□								
U	50,848 (34.2%)	▢▢▢□□	▢▢	□□	▢	□■				□
I	49,745 (36.9%)	▢▢▢	▢▢■	□□■						□■
A	46,831 (42.7%)	No data available from primary informant								
M	46,007 (33.6%)	No data available from primary informant								
J	45,160 (35.1%)	■	■							
E	44,917 (39.8%)									
C	44,858 (38.4%)	▢		□□□□						▢▢□
T	44,373 (34.5%)	▢□□		□■■						
O	42,357 (35.5%)	▢□■■■		■						
V	36,953 (39.7%)	□■		□	▢					
Q	32,674 (38.5%)									□■
K	31,865 (35%)	▢▢▢□□	□□	■	■■			▢		▢
D	28,997 (38.5%)	No data available from primary informant								
B	26,595 (39.8%)	□	▢	□□	□					□□
P	24,912 (35.6%)	▢		▢	▢					□□
F	19,183 (34.3%)									

Legend

▢ Small project (i.e. in one setting within the community or serving fewer than 50 people)

□ Medium sized project (i.e. in more than one location and/or serving 50 or more people)

■ Large project (i.e. serving people throughout the local authority)

NB Wales-wide initiatives such as Walking the Way to Health & lunch clubs are not included

### Examples of innovative health promotion schemes

A few local initiatives stood out as addressing needs that were not being met by the main health promotion initiatives. These were:

• An exercise to music/social club for people who had completed an exercise referral scheme but did not feel comfortable joining public gym sessions.

• A lunch club offering healthy lunches at local pubs and including other activities (talks, demonstrations etc) on a healthy eating theme.

• A project working with older South Asian women to adapt their own traditional recipes to produce healthier meals.

• An educational intervention to promote health and wellbeing and improve independent living skills including nutrition, exercise, budgeting and on-line shopping and banking.

• A substance misuse service for older people offering practical measures such as home safety, falls prevention and smoke alarms as well as help in controlling misuse (mainly of alcohol).

• A comprehensive programme of activities including information, education, social activities physical activities and opportunities for training and volunteering.

### Evidence base and project evaluation

Of 109 regional or local projects, 46 were reported to be informed by evidence such as guidelines, national service framework standards or published research. Most respondents cited WAG strategies (e.g. older people, health, social care and wellbeing) or national service frameworks (older people, coronary heart disease etc) but there was also a range of research, mostly conducted by charitable organisations. Some respondents were aware that projects were underpinned by evidence but were unable to give full details. (Table 5) Information about project evaluation was provided for 47 out of 109 local initiatives but little detail was given. The most commonly used methods were monitoring of user or client numbers and questionnaires to assess user or client satisfaction. Three projects used computer based evaluation tools. Eight projects were being evaluated by independent research groups, but no detail was given regarding the method or rigour of these evaluations.

**Table 5:** Evidence base for projects

**Project type**	**N^o ^of regional or local projects**	**N^o ^said to be evidence based**	**Evidence cited**
Physical activity	42	12	National Service Frameworks for falls, coronary heart disease, healthy living
			WAG Strategy For Older People
			WAG Strategy For Health, Social Care & Wellbeing
			Better Health Strategic Planning Group Action Plan
			Healthy & Active Lifestyles in Wales Task Force (2002)
			Welsh Health Survey 1998
			British Heart Foundation research (no references)
			WAG research (no references)
			Department of Health research (no references)
			Good practice guidance for health (no references)
			Needs assessment
Healthy eating	14	9	National Service Framework for coronary heart disease
			WAG Strategy For Older People
			Evidence from pilot projects
			Success of previous initiatives & other similar projects
Home safety & warmth	19	7	National Service Frameworks for older people (Wales and England)
			WAG Strategy For Older People
			WAG Health Promotion Action Plan For Older People
			Health Development Agency 2002
			Evidence from Royal Society for the Prevention of Accidents (no references)
			Risk assessments of homes
Emotional health	11	4	Published research (no references)
			Guidelines (unspecified)
			10 years past experience of scheme
Health protection	4	3	National Service Frameworks for older people, diabetes, coronary heart disease, heart failure
			WAG Strategy For Older People
			Diabetic Guidelines/Standards Wales
Smoking	1	1	Evidence base for the provision of smoking cessation services
Alcohol	1	1	Evidence of need from referrals
Other	17	9	National Service Frameworks for coronary heart disease, older people
			WAG Health, Social Care & Wellbeing Strategy
			Welsh Health Survey 1998
			St John Ambulance & British Telecom survey of carers 2000
			Carers Outreach study 1999 (no reference)
			Sports Council for Wales focus on increasing physical activity
			Health needs analysis funded by the Big Lottery Fund
			Feasibility study
			Local demographics (30% aged 60+)
			Anecdotal evidence

**Overarching programmes or national campaigns**			

Keep Well This Winter			WAG initiative
			National Service Framework for older people
			Service and Financial Framework guidance
			Welsh Health Circulars
			WAG literature(unspecified)
			National Public Health Service literature (unspecified)
			Evaluation of previous campaigns
Immunisation			Welsh Health Circular (2004) 063 (influenza immunisation)
			Welsh Health Circular (2004) 065 (pneumococcal immunisation)
Luncheon clubs			Evidence from Social Services & Communities First
			Ageing Well UK programme evaluation by Age Concern/WAG
			Food Standards Agency nutrition strategy for Wales
			Local needs assessments
Free swimming for the over 60s			WAG Strategy For Older People
			Local Strategy For Older People
			WAG Strategy For Health, Social Care & Wellbeing
			Better Health Strategic Planning Group Action Plan
			NSF for older people
			Welsh Health Survey (1998)
			Healthy & Active Lifestyles in Wales Task Force (2002)
			Evidence highlighting inactivity in target group (unspecified)
Walking the Way to Health			British Heart Foundation support
			WAG Strategy For Older People
			WAG Strategy For Health, Social Care & Wellbeing
			Climbing Higher (WAG strategy for sport & physical activity)
			Wag Health Promotion Action Plan For Older People
			Better Health Strategic Planning Group Action Plan
			Healthy & Active Lifestyles in Wales Task Force (2002)
			Welsh Health Survey (1998)
			Published research: Morris & Hardman 1997; Ashley A, Bartlett H, Howells K 1997 (incomplete references)
			Countryside Agency research
			Needs assessments
EXTEND			National Service Frameworks for older people, promoting wellbeing & reducing social isolation (standard 8)
			Published research: Windle et al; Skelton D, Dinan S (incomplete references)
			Research (unspecified) supporting hypothesis that EXTEND improves quality of life

### Training needs

Only nine out of 18 PIs completed this section. Unmet training needs were identified for project workers and health professionals. Lack of training for volunteers limited the scope of projects in a variety of settings, particularly those that promoted exercise, healthy eating and emotional support. A dearth of training in the fields of sexual health and alcohol problems was cited as a reason for the absence of health promotion initiatives in these areas. Reasons identified for unmet training needs included under-funding, lack of locally available and appropriate courses and difficulties associated with delivering training in rural areas.

## Discussion

### Summary of results

The amount of health promotion activity reported for older people in Wales varied according to topic area and between local authority areas. Provision was best for physical activity, with three national initiatives and 42 local ones, but local provision was patchy. Healthy eating, home safety and warmth and health protection had far fewer initiatives, as did health protection, which comprised two national immunisation campaigns. One national initiative, Keep Well This Winter, encompassed all the above topic areas, and 19 other local projects had over-arching themes. Smoking and alcohol misuse were poorly targeted at older age groups, and there was no health promotion provision for older people in the area of sexual health. The evaluation arrangements for projects were poorly described. Training needs were poorly reported but shortage of appropriate training appeared to limit the quantity and variety of health promotion activity.

### Study limitations

Whenever possible, we augmented the information obtained from the returned questionnaires with telephone interviews and by using internet searches. However, the depth of inquiry that might have been achieved in surveying a smaller area was to some extent sacrificed for the breadth of this survey. We obtained results for the whole of Wales, and reasonably comprehensive results for 18 of the 22 local authority areas, and we revealed inconsistencies of provision that might not have been apparent from a more detailed survey of selected areas.

In some cases there was difficulty in obtaining fully completed questionnaires due to pressure of work or long-term absence of the primary informants through maternity leave or sickness. In five areas this resulted in the questionnaire being completed by another member of the local health promotion team who, in addition to coping with the additional workload, may not have been as familiar with initiatives for older people. The method and timeframe only allowed for one primary informant to complete the questionnaire, and the research team to pursue any leads given. An additional problem was that secondary informants were not always available and sometimes failed to respond to telephone or email messages, even after reminders. Other possible sources of information such as local voluntary services, charitable organisations, local authorities and public libraries were only approached if contact details had been given by a primary or secondary informant with regard to a specific initiative.

Some local interventions may have been missed by the PIs, as they were asked to report on schemes throughout both health and social care sectors. Interventions in these two sectors may have been categorised differently. For example, some schemes in the social care sector may not have been classified as health promotion. Where PIs had close links, for example with local authorities, Care and Repair, Age Concern and others, often in connection with strategic or funding arrangements, reasonably detailed information was supplied; but where those links did not exist the information was poor.

### Comparison with previous studies

More than half of the local health improvement projects had not been evaluated at all, or the evaluations lacked rigour. This was similar to the findings of another survey of 136 health promotion initiatives for isolated and lonely older people in the North of England [6]. Just under half of these had conducted some form of evaluation or monitoring, but only seven had been evaluated by independent researchers.

### Overlap with other health promotion initiatives

In common with all other age groups, the health of older people is likely to be worse among those living in disadvantaged circumstances, those with disabilities, those who are carers and those from ethnic minority groups. There are health promotion projects in all these areas, many of them provided through Welsh Assembly Government initiatives including the Communities First programme, the Inequalities in Health Fund, the Health Challenge Wales Voluntary Sector Grant Scheme and the New Opportunities (Big Lottery) Fund[5]. Older people may benefit from these projects, as well as those specifically designed for their age-group, provided they are aware of them and consider themselves to be eligible. Standard One of the National Service Framework for Older People in Wales [4] is "rooting out age discrimination" by ensuring that health and social care services are provided regardless of age on the basis of clinical and social need and that age is not used in eligibility criteria or policies to restrict access to and receipt of available services. Initiatives in the fields of smoking cessation, alcohol and sexual health have tended to prioritise younger people; whilst older people are not denied access to existing services, uptake may not be encouraged or services may not be appropriate to their needs. Some services traditionally have not targeted older people and the extent to which this was due to ageist attitudes is unclear; however there is a need to ensure that future services are developed in such a way as to include older people in order to avoid age discrimination by omission.

### Implications for future practice and research

The provision of services across Wales is patchy, but the reasons for this are not clear. Increased efforts to improve the coverage and effectiveness of the interventions known to be useful need to be made, particularly for minority ethnic groups and those in residential care homes where evidence is lacking. Existing projects should be rigorously evaluated using randomised controlled trials, or at least coordinated multi-method evaluations, to establish their effectiveness and cost-effectiveness. New initiatives for older people in the fields of alcohol misuse and sexual health promotion should be commissioned with concurrent evaluation of their acceptability, effectiveness and value for money.

## Ethical Approval

Not required

## Competing interests

The authors declare that they have no competing interests

## Authors' contributions

MH participated in study design, was responsible for data collection and general project management, participated in analysis and interpretation of the data, and prepared the first draft of the paper. NHW was the Principal Investigator, participated in study design, analysis and interpretation of the data, and subsequent re-drafts of the paper. CW was the AWARD project lead, participated in study design and commented on drafts of the paper. All authors read and approved the final manuscript.

## Pre-publication history

The pre-publication history for this paper can be accessed here:



## Supplementary Material

Additional file 1 **Questionnaire about local health promotion programmes and projects for older people.** Click here for file

## References

[B1] Commission of the European Communities (2005). Confronting demographic change: a new solidarity between the generations. Green paper.

[B2] Welsh Assembly Government 2004-based principal population projections for Wales. http://www.statswales.wales.gov.uk/TableViewer/tableView.aspx?ReportId=2511.

[B3] The Swedish National Institute of Public Health (2007). Report: Healthy Ageing: a Challenge for Europe. Report.

[B4] Welsh Assembly Government (2005). National service framework for older people in Wales. Consultation document.

[B5] Welsh Assembly Government (2005). Healthy ageing action plan for Wales.

[B6] Cattan M, White M (1998). Developing evidence based health promotion for older people: a systematic review and survey of health promotion interventions targeting social isolation and loneliness among older people. Internet Journal of Health Promotion.

