# Supplementary material for: A survey of local health promotion initiatives for older people in Wales
Source: BMC Public Health. 2008 Jun 20;8:217. doi: 10.1186/1471-2458-8-217 (PMC2492859; doi:10.1186/1471-2458-8-217)
Supplement: Additional file 1 — Questionnaire about local health promotion programmes and projects for older people. [file 1471-2458-8-217-S1.doc]

**Questionnaire about local health promotion programmes and projects for older people**

This questionnaire asks you about health promotion programmes and projects that are running in your local authority. These may be run by the NHS, local authority, voluntary organisations, the private sector, or a local partnership. Throughout the document, the terms *programme* and *project* are used to mean the following:

**Programme:** A local strategic plan for promoting the health of older people, which includes a number of different projects that focus on a specific topic area or areas. The programme might be a part of the local Health, Social Care and Well-being Strategy, or local Health Alliance Strategy, or other relevant local framework.

**Project:** A component within an overarching programme, or a 'free-standing' project, that focuses on a specific topic area or areas.

**Older people:** means people aged 50 and over.

We are looking for information on programmes and projects that are aimed at older people. These may be aimed exclusively at older people; aimed at the general population but with older people as a specific target group; or aimed at the general population but with a high take up by older people. Please include programmes and projects that are running currently, or are planned within the next two years, with commitment and identified funding from relevant management boards.

Please complete the questionnaire as fully as you can. If you need more space, please use extra sheets, or reply electronically, as the questionnaire will also be sent to you by email. If there are local projects you are aware of, but don't know the full details, please write as much as you can, and include a contact name, address, e-mail address and telephone number for further information.

Sections one to eight are about initiatives that have a topic-specific focus.

The topics are: Physical activity

Healthy eating

Emotional health

Smoking

Alcohol

Sexual health

Home safety & warmth

Health protection

Please use section nine to enter details of other initiatives, including more general health promotion programmes that may be wide-ranging and have multiple aims.

Return the Questionnaire using the pre-paid enclosed envelope to:

FREEPOST RLSY-ASTB-JLUG

Cardiff University

Department of Primary Care & Public Health

North Wales Clinical School

Gwenfro Building

Wrexham Technology Park

LL13 7YP

or e-mail to hendryma@cardiff.ac.uk

**Your Contact Details**

Name: Telephone:

Address e-mail

__________________________________________________________________________________

**Section 1 Projects that promote physical activity**

e.g. encouraging increased activity; participation in sports and exercise.

**Please give details for each project. If there is more than one project in any section, please use the extra sheets, which are supplied at the back of this file, and insert them in the appropriate place. Photocopy more if necessary.**

**Title of Project _**____________________________________________________________________

__________________________________________________________________________________

**Overall aim of project**________________________________________________________________

__________________________________________________________________________________

__________________________________________________________________________________

**Is the project part of an overarching health**

**promotion programme for older people?** Yes/No

If yes, what is the strategic context of the programme? e.g. Health, Social Care and Well-being Strategy or Health Alliance Strategy.

__________________________________________________________________________________

**Organisation(s) involved**

Give name(s) and indicate whether statutory, voluntary or private sector agency.

__________________________________________________________________________________

__________________________________________________________________________________

__________________________________________________________________________________

If one or two of the total number of the above agencies lead the project, please specify which one/s, and give details of the main contact person in each.

__________________________________________________________________________________

__________________________________________________________________________________

__________________________________________________________________________________

**Setting**

e.g. the home; leisure centre or other community setting, primary care, secondary care, intermediate care; residential or nursing home.

__________________________________________________________________________________

__________________________________________________________________________________

**Geographic scope of project**

e.g. is the project throughout the local authority, or is it confined to specific communities or institutions?

__________________________________________________________________________________

__________________________________________________________________________________

**Target group**

Please give a brief description of any defining characteristics of the target group, for e.g. age, gender, social circumstances, health status if relevant (e.g. overweight, at high risk of CHD, has sustained a previous fall)

__________________________________________________________________________________

__________________________________________________________________________________

**Numbers involved**

Please give an estimate of the number of people in the target group who are currently involved in the project…

_______________________________________________________________

…and the maximum capacity of the project.

_______________________________________________________________

**Evidence base**

Is the project informed by evidence, eg guidelines, national service framework standards? Yes/No

If yes, please say what evidence was used.

__________________________________________________________________________________

__________________________________________________________________________________

**Evaluation**

Are processes in place for monitoring or evaluation of the project? Yes/No

If yes, please describe them.

__________________________________________________________________________________

__________________________________________________________________________________

If there are more projects under this topic heading, please use extra sheets from the back of this folder.

**Section 2 Programmes that encourage healthy eating**

e.g. highlight healthy eating messages; improve food shopping or cooking skills; increase access to healthy food.

**Please give details for each project in turn**

**Title of project _**____________________________________________________________________

__________________________________________________________________________________

**Overall aim of project**________________________________________________________________

__________________________________________________________________________________

__________________________________________________________________________________

**Is the project part of an overarching health**

**promotion programme for older people?** Yes/No

If yes, what is the strategic context of the programme?_______________________________________

__________________________________________________________________________________

**Organisation(s) involved**

Give name(s) and indicate whether statutory, voluntary or private sector agency.

__________________________________________________________________________________

__________________________________________________________________________________

__________________________________________________________________________________

If one or two agencies lead the project, please specify which ones and give details of the main contact person in each.

__________________________________________________________________________________

__________________________________________________________________________________

__________________________________________________________________________________

**Setting**

e.g. the home or other community setting; primary care, secondary care, intermediate care; residential or nursing home.

__________________________________________________________________________________

__________________________________________________________________________________

**Geographic scope of project**

e.g. is the initiative throughout Wales, throughout the local authority, or is it confined to specific communities or institutions?

__________________________________________________________________________________

__________________________________________________________________________________

**Target group**

Please give a brief description of any defining characteristics of the target group, for e.g. age, gender, social circumstances, health status if relevant (e.g. overweight, at high risk of CHD, has sustained a previous fall

__________________________________________________________________________________

__________________________________________________________________________________

**Numbers involved**

Please five an estimate of the number of people in the target group who are currently involved in the project…

______________________________________________________________

…and the maximum capacity of the project.

______________________________________________________________

**Evidence base**

Is the project informed by evidence, eg guidelines, national service framework standards? Yes/No

If yes, please say what evidence was used.

__________________________________________________________________________________

__________________________________________________________________________________

**Evaluation**

Are processes in place for monitoring or evaluation of the project? Yes/No

If yes, please describe them.

__________________________________________________________________________________

__________________________________________________________________________________

__________________________________________________________________________________

If there are more projects under this topic heading, please use extra sheets from the back of this folder.

**Section 3 Programmes that promote emotional health**

These might include keeping active and maintaining a social life (other than specific projects described under other topic areas); planning for retirement; befriending and mentoring schemes; and counselling schemes for emotional problems, milder anxiety or depression that can be dealt with in the community, e.g. through primary care or voluntary organisation counselling schemes.

**Please give details for each project in turn**

**Title of project _**____________________________________________________________________

__________________________________________________________________________________

**Overall aim of project**________________________________________________________________

__________________________________________________________________________________

__________________________________________________________________________________

**Is the project part of an overarching health**

**promotion programme for older people?** Yes/No

If yes, what is the strategic context of the programme?_______________________________________

__________________________________________________________________________________

**Organisation(s) involved**

Give name(s) and indicate whether statutory, voluntary or private sector agency.

__________________________________________________________________________________

__________________________________________________________________________________

__________________________________________________________________________________

If one or two agencies lead the project, please specify which ones and give details of the main contact person in each.

__________________________________________________________________________________

__________________________________________________________________________________

__________________________________________________________________________________

**Setting**

e.g. the home, work or other community setting; primary care, secondary care, intermediate care; residential or nursing home.

__________________________________________________________________________________

__________________________________________________________________________________

**Geographic scope of project**

e.g. is the initiative throughout Wales, throughout the local authority, or is it confined to specific communities or institutions?

__________________________________________________________________________________

__________________________________________________________________________________

**Target group**

Please give a brief description of any defining characteristics of the target group, for e.g. age, gender, social circumstances, health status if relevant (e.g. overweight, at high risk of CHD, has sustained a previous fall)

__________________________________________________________________________________

__________________________________________________________________________________

**Numbers involved**

Please give an estimate of the number of people in the target group who are currently involved in the project…

_______________________________________________________________

…and the maximum capacity of the project

_______________________________________________________________

**Evidence base**

Is the project informed by evidence, eg guidelines, national service framework standards? Yes/No

If yes, please say what evidence was used.

__________________________________________________________________________________

__________________________________________________________________________________

**Evaluation**

Are processes in place for monitoring or evaluation of the project? Yes/No

If yes, please describe them.

__________________________________________________________________________________

__________________________________________________________________________________

__________________________________________________________________________________

If there are more projects under this topic heading, please use extra sheets from the back of this folder.

**Section 4 Programmes that reduce the effects of tobacco**

e.g. smoking reduction or cessation; promoting smoke free environments.

**Please give details for each project in turn**

**Title of project _**____________________________________________________________________

__________________________________________________________________________________

**Overall aim of project**________________________________________________________________

__________________________________________________________________________________

__________________________________________________________________________________

**Is the project part of an overarching health**

**promotion programme for older people?** Yes/No

If yes, what is the strategic context of the programme?_______________________________________

__________________________________________________________________________________

**Organisation(s) involved**

Give name(s) and indicate whether statutory, voluntary or private sector agency.

__________________________________________________________________________________

__________________________________________________________________________________

__________________________________________________________________________________

If one or two agencies lead the project, please specify which ones and give details of the main contact person in each.

__________________________________________________________________________________

__________________________________________________________________________________

__________________________________________________________________________________

Setting

e.g. the home or other community setting; primary care, secondary care, intermediate care; residential or nursing home.

__________________________________________________________________________________

__________________________________________________________________________________

**Geographic scope of project**

e.g. is the initiative throughout Wales, throughout the local authority, or is it confined to specific communities or institutions?

__________________________________________________________________________________

__________________________________________________________________________________

**Target group**

Please give a brief description of any defining characteristics of the target group, for e.g. age, gender, social circumstances, health status if relevant (e.g. overweight, at high risk of CHD, has sustained a previous fall)

__________________________________________________________________________________

__________________________________________________________________________________

**Numbers involved**

Please give an estimate of the number of people in the target group who are currently involved in the project…

______________________________________________________________

…and the maximum capacity of the project

______________________________________________________________

**Evidence base**

Is the project informed by evidence, eg guidelines, national service framework standards? Yes/No

If yes, please say what evidence was used.

__________________________________________________________________________________

__________________________________________________________________________________

**Evaluation**

Are processes in place for monitoring or evaluation of the project? Yes/No

If yes, please describe them.

__________________________________________________________________________________

__________________________________________________________________________________

__________________________________________________________________________________

If there are more projects under this topic heading, please use extra sheets from the back of this folder.

**Section 5 Programmes that promote sensible drinking**

e.g. identifying alcohol misuse; helping to give up or reduce alcohol consumption; facilitating access to services.

**Please give details for each project in turn**

**Title of project _**____________________________________________________________________

__________________________________________________________________________________

**Overall aim of project**________________________________________________________________

__________________________________________________________________________________

__________________________________________________________________________________

**Is the project part of an overarching health**

**promotion programme for older people?** Yes/No

If yes, what is the strategic context of the programme?_______________________________________

__________________________________________________________________________________

**Organisation(s) involved**

Give name(s) and indicate whether statutory, voluntary or private sector agency.

__________________________________________________________________________________

__________________________________________________________________________________

__________________________________________________________________________________

If one or two agencies lead the project, please specify which ones and give details of the main contact person in each.

__________________________________________________________________________________

__________________________________________________________________________________

__________________________________________________________________________________

**Setting**

e.g. primary care, secondary care, intermediate care; residential or nursing care; the home or other community setting.

__________________________________________________________________________________

__________________________________________________________________________________

__________________________________________________________________________________

**Geographic scope of project**

e.g. is the initiative throughout Wales, throughout the local authority, or is it confined to specific communities or institutions?

__________________________________________________________________________________

__________________________________________________________________________________

**Target group**

Please give a brief description of any defining characteristics of the target group, for e.g. age, gender, social circumstances, health status if relevant (e.g. overweight, at high risk of CHD, has sustained a previous fall)

__________________________________________________________________________________

__________________________________________________________________________________

**Numbers involved**

Please give an estimate of the number of people in the target group who are currently involved in the project…

______________________________________________________________

…and the maximum capacity of the project

______________________________________________________________

**Evidence base**

Is the project informed by evidence, eg guidelines, national service framework standards? Yes/No

If yes, please say what evidence was used.

__________________________________________________________________________________

__________________________________________________________________________________

**Evaluation**

Are processes in place for monitoring or evaluation of the project? Yes/No

If yes, please describe them.

__________________________________________________________________________________

__________________________________________________________________________________

__________________________________________________________________________________

If there are more projects under this topic heading, please use extra sheets from the back of this folder.

**Section 6 Programmes that promote sexual health**

e.g. facilitating access to sexual health services, promoting the right to privacy for people in residential care, combating ageist attitudes.

**Please give details for each project in turn**

**Title of project _**____________________________________________________________________

__________________________________________________________________________________

**Overall aim of project**________________________________________________________________

__________________________________________________________________________________

__________________________________________________________________________________

**Is the project part of an overarching health**

**promotion programme for older people?**  Yes/No

If yes, what is the strategic context of the programme?_______________________________________

__________________________________________________________________________________

**Organisation(s) involved**

Give name(s) and indicate whether statutory, voluntary or private sector agency.

__________________________________________________________________________________

__________________________________________________________________________________

__________________________________________________________________________________

If one or two agencies lead the project, please specify which ones and give details of the main contact person in each.

__________________________________________________________________________________

__________________________________________________________________________________

__________________________________________________________________________________

**Setting**

e.g. primary care, secondary care, intermediate care; residential or nursing care; the home or other community setting.

__________________________________________________________________________________

__________________________________________________________________________________

**Geographic scope of project**

e.g. is the initiative throughout Wales, throughout the local authority, or is it confined to specific communities or institutions?

__________________________________________________________________________________

__________________________________________________________________________________

**Target group**

Please give a brief description of any defining characteristics of the target group, for e.g. age, gender, social circumstances, health status if relevant (e.g. overweight, at high risk of CHD, has sustained a previous fall)

__________________________________________________________________________________

__________________________________________________________________________________

**Numbers involved**

Please give an estimate of the number of people in the target group who are currently involved in the project…

______________________________________________________________

…and the maximum capacity of the project

______________________________________________________________

**Evidence base**

Is the project informed by evidence, eg guidelines, national service framework standards? Yes/No

If yes, please say what evidence was used.

__________________________________________________________________________________

__________________________________________________________________________________

**Evaluation**

Are processes in place for monitoring or evaluation of the project? Yes/No

If yes, please describe them.

__________________________________________________________________________________

__________________________________________________________________________________

__________________________________________________________________________________

If there are more projects under this topic heading, please use extra sheets from the back of this folder.

**Section 7 Programmes that promote home safety and warmth**

e.g. grants and assistance with home insulation, maintenance, safety of appliances, home security.

**Please give details for each project in turn**

**Title of project _**____________________________________________________________________

__________________________________________________________________________________

**Overall aim of project**________________________________________________________________

__________________________________________________________________________________

__________________________________________________________________________________

**Is the project part of an overarching health**

**promotion programme for older people?** Yes/No

If yes, what is the strategic context of the programme?_______________________________________

__________________________________________________________________________________

**Organisation(s) involved**

Give name(s) and indicate whether statutory, voluntary or private sector agency.

__________________________________________________________________________________

__________________________________________________________________________________

__________________________________________________________________________________

If one or two agencies lead the project, please specify which ones and give details of the main contact person in each.

__________________________________________________________________________________

__________________________________________________________________________________

________________________________________________________________________________

**Setting**

e.g. primary care, secondary care, intermediate care; residential or nursing care; the home or other community setting.

__________________________________________________________________________________

__________________________________________________________________________________

**Geographic scope of project**

e.g. is the initiative throughout Wales, throughout the local authority, or is it confined to specific communities or institutions?

__________________________________________________________________________________

__________________________________________________________________________________

**Target group**

Please give a brief description of any defining characteristics of the target group, for e.g. age, gender, social circumstances, health status if relevant (e.g. overweight, at high risk of CHD, has sustained a previous fall)

__________________________________________________________________________________

__________________________________________________________________________________

**Numbers involved**

Please give an estimate of the number of people in the target group who are currently involved in the project…

______________________________________________________________

…and the maximum capacity of the project

______________________________________________________________

**Evidence base**

Is the project informed by evidence, eg guidelines, national service framework standards? Yes/No

If yes, please say what evidence was used.

__________________________________________________________________________________

__________________________________________________________________________________

**Evaluation**

Are processes in place for monitoring or evaluation of the project? Yes/No

If yes, please describe them.

__________________________________________________________________________________

__________________________________________________________________________________

__________________________________________________________________________________

If there are more projects under this topic heading, please use extra sheets from the back of this folder.

**Section 8 Health protection programmes**

e.g. increasing immunisation rates; general health screening; raising awareness of health issues.

**Please give details for each project in turn**

**Title of project _**____________________________________________________________________

__________________________________________________________________________________

**Overall aim of project**________________________________________________________________

__________________________________________________________________________________

__________________________________________________________________________________

**Is the project part of an overarching health**

**promotion programme for older people?** Yes/No

If yes, what is the strategic context of the programme?_______________________________________

__________________________________________________________________________________

**Organisation(s) involved**

Give name(s) and indicate whether statutory, voluntary or private sector agency.

__________________________________________________________________________________

__________________________________________________________________________________

__________________________________________________________________________________

If one or two agencies lead the project, please specify which ones and give details of the main contact person in each.

__________________________________________________________________________________

__________________________________________________________________________________

__________________________________________________________________________________

**Setting**

e.g. primary care, secondary care, intermediate care; residential or nursing care; the home or other community setting.

__________________________________________________________________________________

__________________________________________________________________________________

**Geographic scope of project**

e.g. is the initiative throughout Wales, throughout the local authority, or is it confined to specific communities or institutions?

__________________________________________________________________________________

__________________________________________________________________________________

**Target group**

Please give a brief description of any defining characteristics of the target group, for e.g. age, gender, social circumstances, health status if relevant (e.g. overweight, at high risk of CHD, has sustained a previous fall)

__________________________________________________________________________________

__________________________________________________________________________________

**Numbers involved**

Please give an estimate of the number of people in the target group who are currently involved in the project…

______________________________________________________________

…and the maximum capacity of the project

______________________________________________________________

**Evidence base**

Is the project informed by evidence, eg guidelines, national service framework standards? Yes/No

If yes, please say what evidence was used.

__________________________________________________________________________________

__________________________________________________________________________________

**Evaluation**

Are processes in place for monitoring or evaluation of the project? Yes/No

If yes, please describe them.

__________________________________________________________________________________

__________________________________________________________________________________

__________________________________________________________________________________

If there are more projects under this topic heading, please use extra sheets from the back of this folder.

**Section 9 Other projects**

This includes any other projects not already described including those with a wider range of activities, rather than focused on specific topic areas.

**Please give details for each project in turn**

**Title of project _**____________________________________________________________________

__________________________________________________________________________________

**Overall aim of project**________________________________________________________________

__________________________________________________________________________________

__________________________________________________________________________________

**Is the project part of an overarching health promotion programme for older people?**

Yes/No

If yes, what is the strategic context of the programme?_______________________________________

__________________________________________________________________________________

**Organisation(s) involved**

Give name(s) and indicate whether statutory, voluntary or private sector agency.

__________________________________________________________________________________

__________________________________________________________________________________

__________________________________________________________________________________

If one or two agencies lead the project, please specify which ones and give details of the main contact person in each.

__________________________________________________________________________________

__________________________________________________________________________________

__________________________________________________________________________________

**Setting**

e.g. primary care, secondary care, intermediate care; residential or nursing care; the home or other community setting.

__________________________________________________________________________________

__________________________________________________________________________________

**Geographic scope of project**

e.g. is the initiative throughout Wales, throughout the local authority, or is it confined to specific communities or institutions?

__________________________________________________________________________________

__________________________________________________________________________________

**Target group**

Please give a brief description of any defining characteristics of the target group, for e.g. age, gender, social circumstances, health status if relevant (e.g. overweight, at high risk of CHD, has sustained a previous fall)

__________________________________________________________________________________

__________________________________________________________________________________

**Numbers involved**

Please give an estimate of the number of people in the target group who are currently involved in the project…

______________________________________________________________

…and the maximum capacity of the project

______________________________________________________________

**Evidence base**

Is the project informed by evidence, eg guidelines, national service framework standards? Yes/No

If yes, please say what evidence was used.

__________________________________________________________________________________

__________________________________________________________________________________

**Evaluation**

Are processes in place for monitoring or evaluation of the project? Yes/No

If yes, please describe them.

__________________________________________________________________________________

__________________________________________________________________________________

__________________________________________________________________________________

If there are more projects under this topic heading, please use extra sheets from the back of this folder.

**Section 10 Training needs**

This section applies to all the projects described in this questionnaire.

In the course of developing or running these projects, have you become

aware of any training needs in relation to health promotion for older people? Yes/No

If yes, what were they and how have they been met?

___________________________________________________________________________

___________________________________________________________________________

___________________________________________________________________________

___________________________________________________________________________

___________________________________________________________________________

___________________________________________________________________________

___________________________________________________________________________

___________________________________________________________________________

If the training needs could not be met, please give reasons. e.g. were no appropriate courses available, or was there a lack of funding to pay for training?

___________________________________________________________________________

___________________________________________________________________________

___________________________________________________________________________

___________________________________________________________________________

___________________________________________________________________________

___________________________________________________________________________

___________________________________________________________________________

___________________________________________________________________________

**Thank you for completing this questionnaire**
